# Supplementary figures and images for: A systematic review and meta-analysis of the prevalence of childhood undernutrition in North Africa
Source: PLoS One. 2023 Apr 6;18(4):e0283685. doi: 10.1371/journal.pone.0283685 (PMC10079122; doi:10.1371/journal.pone.0283685)

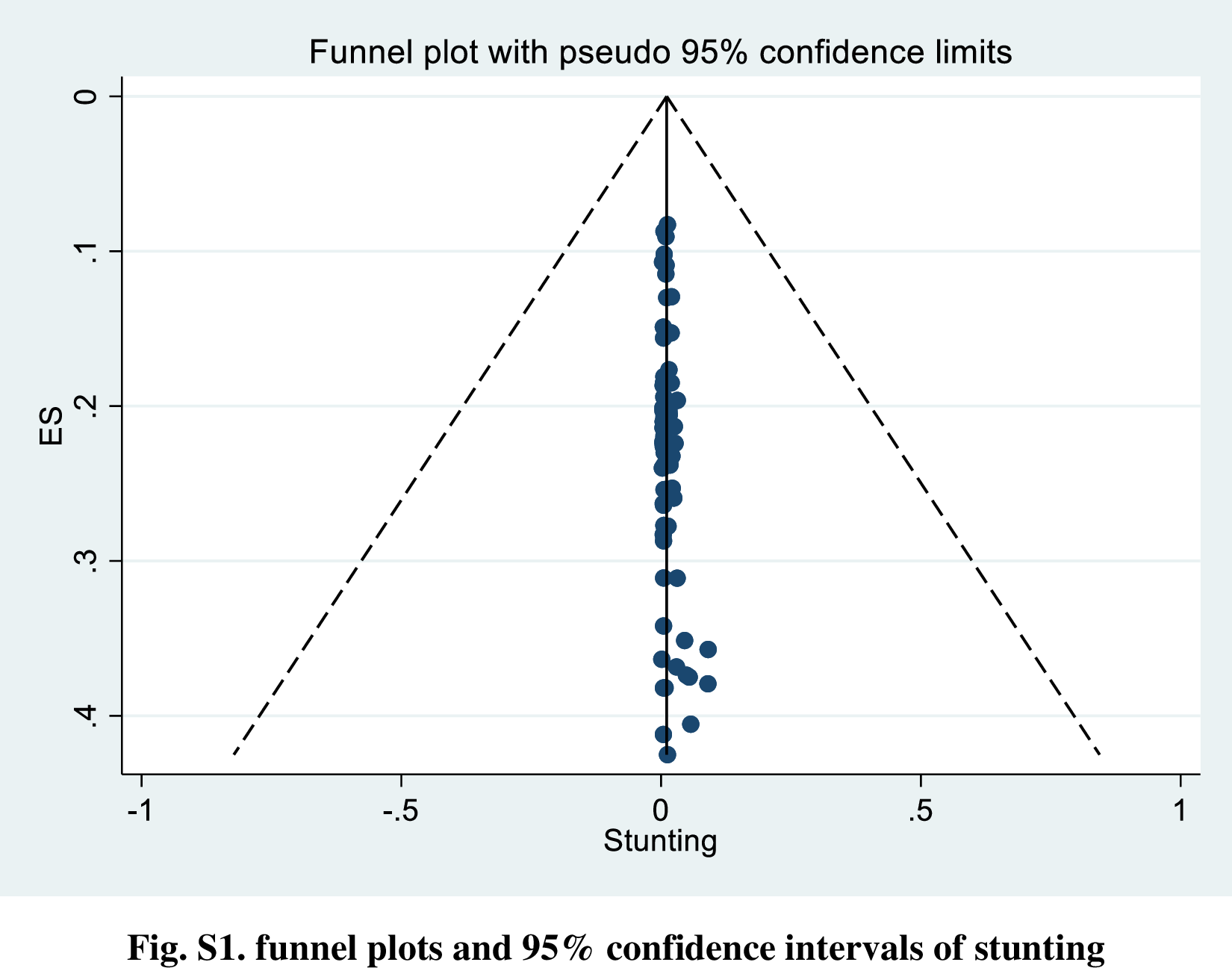

Supplement: S1 Fig — (TIF) [file pone.0283685.s001.tif]

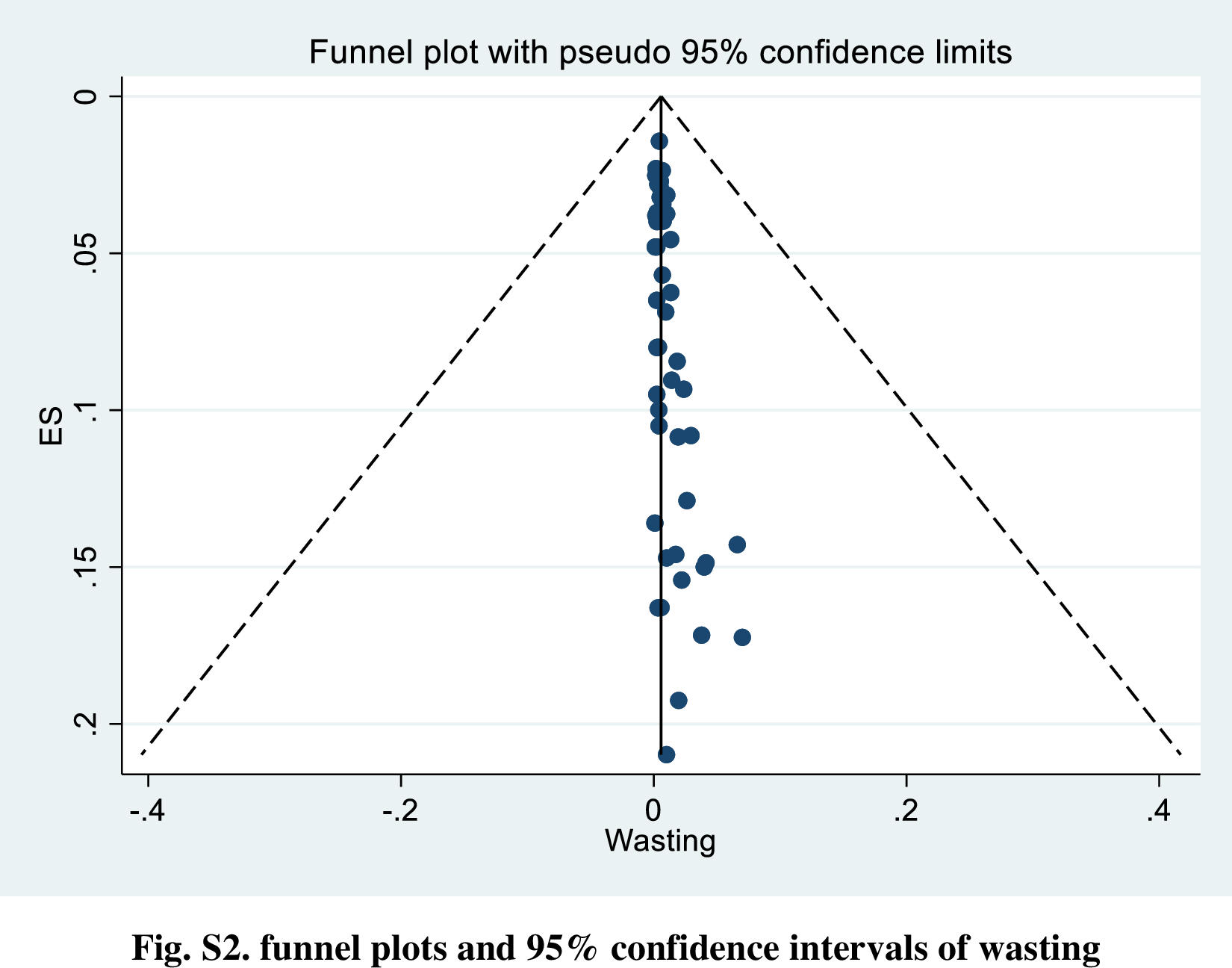

Supplement: S2 Fig — (TIF) [file pone.0283685.s002.tif]

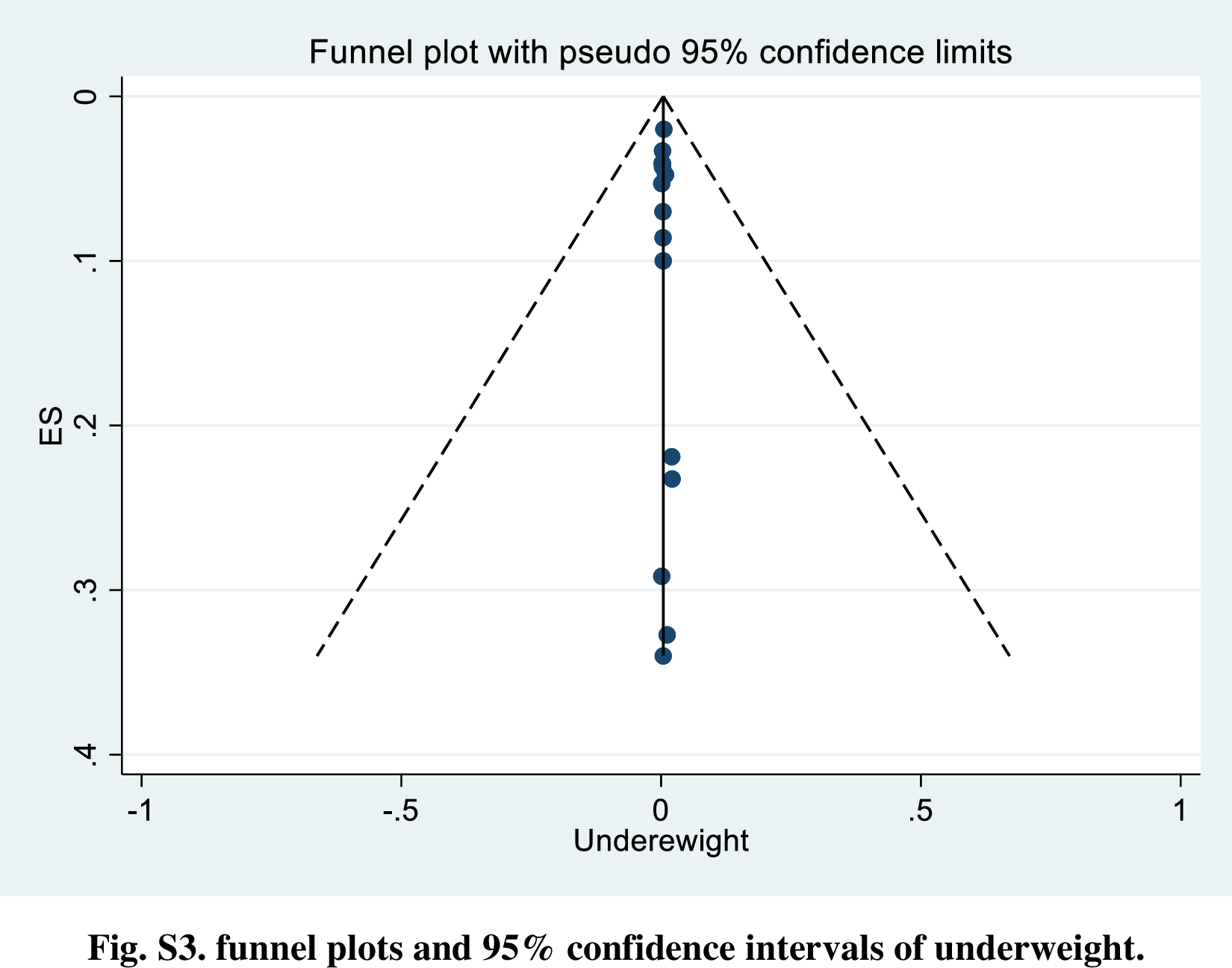

Supplement: S3 Fig — (TIF) [file pone.0283685.s003.tif]

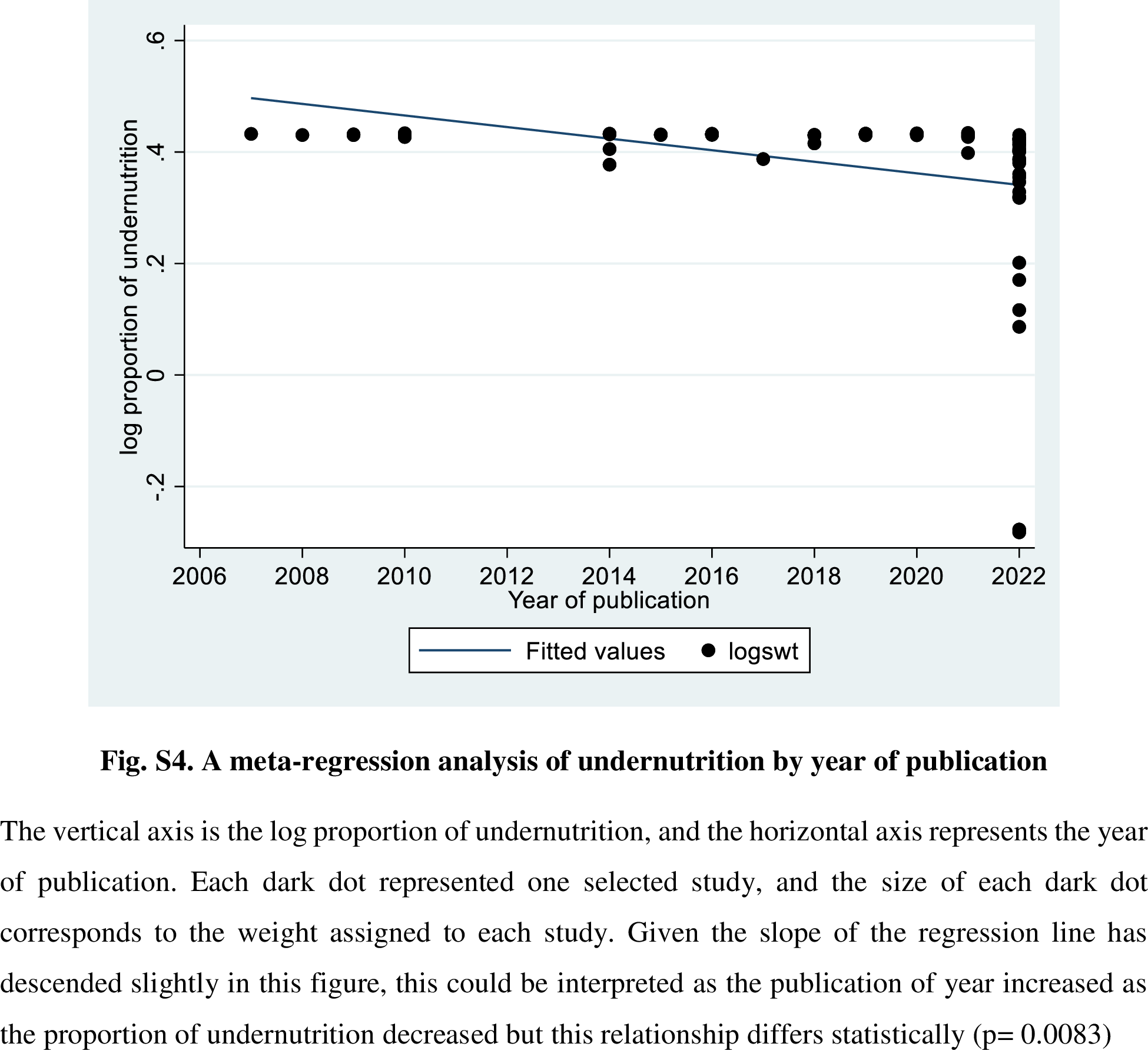

Supplement: S4 Fig — The vertical axis is the log proportion of undernutrition, and the horizontal axis represents the year of publication. Each dark dot represented one selected study, and the size of each dark dot corresponds to the weight assigned to each study. Given the slope of the regression line has descended slightly in this figure, this could be interpreted as the publication of year increased as the proportion of undernutrition decreased but this relationship differs statistically (p = 0.0083). (TIF) [file pone.0283685.s004.tif]
