# Supplementary material for: A systematic review and meta-analysis of the prevalence of childhood undernutrition in North Africa
Source: PLoS One. 2023 Apr 6;18(4):e0283685. doi: 10.1371/journal.pone.0283685 (PMC10079122; doi:10.1371/journal.pone.0283685)
Supplement: S3 Table — (DOCX) [file pone.0283685.s007.docx]

| **Author; year** | **1. Was the sample frame appropriate to address the target population?** | **2. Were study participants sampled in an appropriate way?** | **3. Was the sample size adequate?** | **4. Were the study subjects and the setting described in detail?** | **5. Was the data analysis conducted with sufficient coverage of the identified sample?** | **6. Were valid methods used for the identification of the condition?** | **7. Was the condition measured in a standard, reliable way for all participants?** | **8. Was there appropriate statistical analysis?** | **9. Was the response rate adequate, and if not, was the low response rate managed appropriately?** | **Quality assessment score** |
| --- | --- | --- | --- | --- | --- | --- | --- | --- | --- | --- |
| Abdalla et al. 2009 | 1 | 0 | 0 | 1 | 1 | 1 | 1 | 0 | 1 | 6 |
| Abu-Manga et al. 2021 | 1 | 1 | 1 | 1 | 1 | 1 | 0 | 1 | 1 | 8 |
| Aitsi-Selmi 2014 | 1 | 1 | 1 | 1 | 1 | 1 | 0 | 1 | 1 | 8 |
| Almasi et al. 2019 | 1 | 1 | 1 | 0 | 1 | 1 | 0 | 0 | 1 | 6 |
| Barouaca 2012 | 1 | 1 | 1 | 0 | 1 | 1 | 0 | 0 | 1 | 6 |
| Dahab et al 2020 | 1 | 1 | 0 | 1 | 1 | 1 | 0 | 1 | 1 | 7 |
| Elsary 2017 | 1 | 1 | 0 | 1 | 1 | 1 | 0 | 1 | 1 | 7 |
| El-Taguri et al. 2009 | 1 | 1 | 1 | 1 | 1 | 1 | 0 | 1 | 1 | 8 |
| El Taguri et al. 2008 | 1 | 1 | 1 | 1 | 1 | 1 | 0 | 1 | 1 | 8 |
| Fagbamigbe et al. 2020 | 1 | 1 | 1 | 1 | 1 | 1 | 0 | 1 | 1 | 8 |
| Figueroa and Kurdi 2019 | 1 | 1 | 1 | 0 | 0 | 1 | 0 | 0 | 1 | 6 |
| Ghattas et al. 2020 | 1 | 1 | 1 | 1 | 1 | 1 | 0 | 1 | 1 | 8 |
| Kavle et al. 2015 | 1 | 1 | 1 | 1 | 1 | 1 | 0 | 1 | 1 | 8 |
| Kerac et al. 2019 | 1 | 1 | 1 | 1 | 0 | 1 | 0 | 1 | 1 | 7 |
| Kiarie et al. 2021 | 1 | 1 | 0 | 1 | 1 | 1 | 0 | 1 | 1 | 7 |
| Mberu et al. 2016 | 1 | 1 | 1 | 0 | 1 | 1 | 0 | 0 | 1 | 6 |
| Musa et al. 2014 | 1 | 1 | 0 | 1 | 1 | 0 | 1 | 0 | 1 | 6 |
| Nikooyeh et al. 2022 | 1 | 0 | 0 | 0 | 1 | 1 | 0 | 1 | 1 | 5 |
| Ozaltin et al. 2010 | 1 | 1 | 1 | 1 | 1 | 1 | 0 | 1 | 1 | 8 |
| Rico et al. 2010 | 1 | 1 | 1 | 1 | 1 | 1 | 0 | 1 | 1 | 8 |
| Seedhom et al. 2014 | 1 | 1 | 0 | 1 | 1 | 1 | 0 | 1 | 1 | 6 |
| Shaker-Berbari et al 2021 | 1 | 1 | 1 | 1 | 1 | 1 | 0 | 1 | 1 | 8 |
| Sharaf and Rashad 2016 | 1 | 1 | 1 | 1 | 1 | 1 | 0 | 0 | 1 | 6 |
| Sulaiman et al. 2018 | 1 | 1 | 1 | 1 | 1 | 1 | 0 | 1 | 1 | 8 |
| Tzioumis et al. 2016 | 1 | 1 | 1 | 1 | 1 | 1 | 0 | 0 | 1 | 7 |
| Winskill et al. 2021 | 1 | 1 | 1 | 1 | 1 | 1 | 0 | 1 | 1 | 8 |
| Zotarelli et al. 2007 | 1 | 1 | 1 | 1 | 1 | 1 | 0 | 1 | 1 | 8 |

**S3 Table. Quality assessment score**
