# Supplementary material for: A systematic review and meta-analysis of the prevalence of childhood undernutrition in North Africa
Source: PLoS One. 2023 Apr 6;18(4):e0283685. doi: 10.1371/journal.pone.0283685 (PMC10079122; doi:10.1371/journal.pone.0283685)
Supplement: S4 Table — (DOCX) [file pone.0283685.s008.docx]

**S4 Table. Prevalence of undernutrition among children under five in North Africa**

| **Country** | **Authors** | **Year of data collection** | **Stunting %** | **Wasting %** | **Under weight** |
| --- | --- | --- | --- | --- | --- |
| **Egypt** | Almasi et al. 2019 | 2014 | 22.3 | 9.5 | - |
|  | Aitsi-Selmi 2014 | 1992-95 | 26.4 | - | - |
|  |  | 2005-08 | 20.3 | - | - |
|  | Elsary 2017 | 2014 | 18.5 | 19.3 | 23.2 |
|  | Fagbamigbe et al. 2020 | 2014 | - | 3.8 | - |
|  | Figueroa and Kurdi 2019 | 2014 | 20.1 | - | - |
|  |  | 2008 | 31.1 | - | - |
|  |  | 2005 | 28.3 | - | - |
|  | Ghattas et al. 2020 | 2014 | 22.4 | - | - |
|  | Kavle et al. 2015 | 2008 | 25.4 | - | - |
|  |  | 2005 | 22.6 | - | - |
|  | Kerac et al. 2019 | 2014 | - | 14.7 | - |
|  | Mberu et al. 2016 | 2003 | 15.6 | 4.0 | 8.6 |
|  | Nikooyeh et al.  2022 | 2020 | 19.7 | 13 | - |
|  |  | 2015 | 21.4 | 10.9 | - |
|  |  | 2010 | 23.3 | 9.1 | - |
|  |  | 2005 | 25.5 | 6.9 | - |
|  |  | 2000 | 27.8 | 5.73 | - |
|  | Ozaltin et al. 2010 | 2000-2008 | 24 | 4.8 | 5.3 |
|  | Rico et al. 2010 | 2005 | 25.4 | - | - |
|  | Seedhom et al. 2014 | 2014 | 20.3 | 1.4 | 2 |
|  | Shaker-Berbari et al. 2021 | 2014 | 21 | 8.0 | - |
|  | Sharaf and Rashad 2016 | 2014 | 20 | - | - |
|  | Tzioumis et al. 2016 | 2008 | 28.7 | - | - |
|  |  | 2005 | 26.3 | - | - |
|  |  | 2003 | 19.4 | - | - |
|  |  | 2000 | 22.6 | - | - |
|  |  | 1995 | 34.2 | - | - |
|  |  | 1992 | 27.7 | - | - |
|  | Winskill et al. 2021 | 2014 | - | 8 | - |
|  | Zottarelli et al. 2007 | 2000 | 18.67 | 2.52 | 4.06 |
| **Sudan** | Abdalla et al. 2009 | 2003 | - | 9.3% | - |
|  | Abu-Manga et al. 2021 | 2019 | 36.35 | 13.6 | 29.16 |
|  | (Almasi et al. 2019) | 2014 | 38.2 | 16.3 | - |
|  | Dahab et al. 2020 | 2014 | - | - | 34.0 |
|  | Ghattas et al. 2020 | 2014 | 41.2 | - | - |
|  | Kiarie et al. 2021 | 2018 | 23.8 | 2.3 | 4.8 |
|  | Musa et al.  2014 | 2014 | 24.9 | 21.1 | 15.4 |
|  | Nikooyeh et al. 2022 | 2020 | 36.8 | 18.2 | - |
|  |  | 2015 | 37.7 | 16.7 | - |
|  |  | 2010 | 37 | 15.4 | - |
|  |  | 2005 | 37 | 15.4 | - |
|  |  | 2000 | 36.9 | 14.2 | - |
|  | Shaker-Berbari et al 2021 | 2014 | 38.2 | 16.3 | - |
|  | Sulaiman et al. 2018 | 2014 | 42.5 | 21 | 32.7 |
| **Libya** | (Almasi et al. 2019) | 2007 | 21 | 6.5 | - |
|  | El Taguri et al. 2009 | 2003 | 18.5 | 4.8 | - |
|  | El Taguri et al. 2008 | 1995 | 20.7 | 3.7 | 4.3 |
|  | Nikooyeh et al. 2022 | 2020 | 40.1 | 14.2 | - |
|  |  | 2015 | 35.4 | 10.9 | - |
|  |  | 2010 | 31 | 8.3 | - |
|  |  | 2005 | 25.9 | 6.3 | - |
|  |  | 2000 | 22.2 | 4.7 | - |
| **Algeria** | Ghattas et al. 20120 | 2013 | 10.7 | - | - |
| **Morocco** | Almasi et al. 2019 | 2011 | 14.9 | 2.3 | - |
|  | El Taguri et al. 2009 | 2003 | 22 | 10.2 | - |
|  | Ghattas et al. 2020 | 2003 | 23.8 |  |  |
|  | Nikooyeh et al. 2022 | 2020 | 13 | 3.2 | - |
|  |  | 2015 | 15.4 | 3.6 | - |
|  |  | 2010 | 17.7 | 3.5 | - |
|  |  | 2005 | 20.5 | 3.9 | - |
|  |  | 2000 | 23.7 | 3.9 | - |
|  | Ozaltin et al. 2010 | 2003-04 | 21.9 | 10 | 10 |
|  | Barouaca 2012 | 2004 | 18.1 | - | 7 |
|  |  | 1997 | 23 | - | 4.1 |
|  |  | 1992 | 22.6 | - | 3.3 |
| **Tunisia** | Almasi et al. 2019) | 2012 | 10.2 | 2.8 | - |
|  | Ghattas et al. 2020 | 2012 | 8.7 | - | - |
|  | Nikooyeh et al. 2022 | 2020 | 8.3 | 2.4 | - |
|  |  | 2015 | 9.1 | 2.7 | - |
|  |  | 2010 | 10.9 | 2.9 | - |
|  |  | 2005 | 11.5 | 3.2 | - |
|  |  | 2000 | 13 | 3.6 | - |
