# Supplementary material for: A systematic review and meta-analysis of the prevalence of childhood undernutrition in North Africa
Source: PLoS One. 2023 Apr 6;18(4):e0283685. doi: 10.1371/journal.pone.0283685 (PMC10079122; doi:10.1371/journal.pone.0283685)
Supplement: Attachment — Submitted filename: Response to Reviewers.docx [file pone.0283685.s009.docx]

**Response to editors**

30 December 2022

**Manuscript ID:** PONE-D-22-26443

“A systematic review and meta-analysis of the prevalence of childhood undernutrition in North Africa” (**Authors:** **Elmighrabi, Fleming, Dhami, Elmabsout & Agho**)

**Dear Editor-in-chief,**

Thank you for allowing us to Dham revise and re-submit our manuscript. We are incredibly grateful for your insightful feedback that has strengthened our manuscript.

We have addressed all the comments raised by the reviewers in the revised version of the manuscript. Where we have a different opinion from the reviewers, we have clarified our position. All changes made to the revised manuscript have been highlighted in yellow. Below, we provide a point-by-point response to the comments and, where possible, linked our responses to the appropriate sections in the revised manuscript. We hope that the revisions made have properly addressed the concerns and that our revised manuscript is accepted for publication in the journal.

**Response to the journal requirements**

1. Please ensure that your manuscript meets PLOS ONE's style requirements, including those for file naming. The PLOS ONE style templates can be found at 
[https://journals.plos.org/plosone/s/file?id=wjVg/PLOSOne_formatting_sample_main_body.pdf](https://protect-au.mimecast.com/s/rbU1CjZ1oBSAKWQOH7nPkH?domain=journals.plos.org) and 
[https://journals.plos.org/plosone/s/file?id=ba62/PLOSOne_formatting_sample_title_authors_affiliations.pdf](https://protect-au.mimecast.com/s/erzjCk81pgC48xzwhJs-SO?domain=journals.plos.org)

***Authors response:*** Sorted out and every requirement considered

2. Please amend the manuscript submission data (via Edit Submission) to include authors Catharine A.K. Fleming, Mansi Vijaybhai Dham, Ali 
Ateia Elmabsout and Kingsley E. Agho.

***Authors response:*** *sorted out*

3. Please include a copy of Table 2 which you refer to in your text on page 15.

***Authors response:*** *sorted out as it is not table 2 but S4 Table*

4. Please include captions for your Supporting Information files at the end of your manuscript, and update any in-text citations to match accordingly. Please see our Supporting Information guidelines for more information: [http://journals.plos.org/plosone/s/supporting-information](https://protect-au.mimecast.com/s/CmreClx1q0fk0QNMSYZQWk?domain=journals.plos.org).

***Authors response:*** *sorted out (Lines 618-623)*

5. We note that this manuscript is a systematic review or meta-analysis; our author guidelines therefore require that you use PRISMA guidance to help improve reporting quality of this type of study. Please upload copies of the completed PRISMA checklist as Supporting Information with a file name “PRISMA checklist”.

***Authors response:*** *sorted out S1 and S2 Files for manuscript and abstract*

**Reviewer #1:**

**Response to the reviewers**

**Reviewer #1:** The paper 'A systematic review and meta-analysis of the prevalence of childhood undernutrition in North Africa' is written well, analysis is made in very good way. But need some minor correction, which are as follow.
**1. Citation is not in correct manner.**

**The authors' response:** *sorted out and changed to Vancouver style*
**2. Funnel plot if you made country-wide, heterogeneity can be easy assessed.**

**The authors' response:** *We agreed with the author’s point, but a country-wide plot would nullify the aim of the paper, which is to ‘systematically review and meta-analysed the prevalence of childhood undernutrition in North Africa.'*

**Reviewer #2:**

**Reviewer #2:** Well-done on your effort with this review of child undernutrition in North Africa. See below my comments to make your study stronger.
Abstract
**Review this sentence in the abstract for grammatical error "Therefore, we aimed to systematically review and meta-analysis the prevalence of undernutrition .........".
The authors' response*: sorted out.*** *“Therefore, we aimed to systematically review and meta-analyse (line 25)*
***What is the significance of this statement: "More than one in ten children in Algeria and Tunisia had stunted growth"***
**The authors' response:** It still represents a significant value as in the SDGs 2017, in the target 2.2 by 2030 *end* all *forms of malnutrition, which would not be in North African countries as the burdens of undernutrition are increasing instead of decreasing as stated in WHO and UNICEF report 2021.*
**Introduction**
Overall: The introduction section appears disjointed with lots of grammatical errors which makes the narrative hard to comprehend. The authors attempt making some points, but the points do not seem to flow logically across the paragraphs. I strongly advise a revision of the entire introduction section so it flows logically.
See below more specific comments.
**In lines 65 - 66, what do you mean by "Burdens of undernutrition range from high to remarkably high among children in North African countries"?. How is "high to remarkably high" quantified? any reference? Please rephrase the sentence and include a reference.**
**The authors' response*: sorted out.*** *Burdens of undernutrition range from high to remarkably high among children in North African countries, as stunting affects over 35% of under-five children in Libya and Sudan and 22. 3% in Egypt, while 6.6 % of children in the entire region suffer from wasting (1). (Lines 56-58)*
**Review this sentence in lines 69 - 70 for grammatical error: "It increases morbidity and mortality by exacerbates disease and makes children more susceptible to illness".**
**The authors' response: *sorted out.*** *Diseases and illnesses (line 61)*
**From lines 76 - 81, the dive into the discussion on health care cost was quite sudden and unrelated and does not add to nor continue the preceding discussion. It appears irrelevant to the on-going discussion.**
**The authors' response:** *sorted out. (Lines 67and 68)*

**Review this sentence in lines 81 - 82 for grammatical error: "Therefore, eliminating all forms of undernutrition is crucial, and requires investment interventions of collecting, analyzing, and quality data, which is the key....."
The authors' response: *sorted out.*** *Therefore, eliminating all forms of undernutrition is crucial and requires investments in collecting and analyzing quality data, which is the key to achieving SDGs 1, 2 and 3 and imperative to tracking the progress of essential government initiatives (lines 72-75)*

**What do you mean in lines 92 - 95 by: "Apart from the child age , no studies were included on Libya, Algeria, Tunisia, or Western Sahara, even though they were a part of the study. Only four included studies focused on Egypt, two on Sudan, and one on Morocco". What study are you referring to here? This sentence is quite confusing because it does not follow a preceding line of thought and providing a reference is not enough.**

**The authors' response*: sorted out.*** *Previously, a study examined 24 countries in the Middle Eastern and North African regions for the only prevalence of stunting in children aged 2-18 (19). Aside from including the wide range of child ages, which affects the results validity and generalizability, no information was included about Libya, Algeria or Tunisia despite their participation in the study (19). (Lines 82-85)*

**In lines 98 - 99 you wrote: "However, this study was limited in several ways: First, the authors indicated that they conducted literature search from PubMed and google scholar, but only reported prevalence from published reports". How is this a limitation? Are reports not found in PubMed and google scholar?**
**The authors' response**: *sorted out (lines 86-104)*
In lines 99 - 100 you wrote: "Secondly the authors’ literature search was restricted to the period 2019-2020;". How is this a limitation?
**The authors' response:** *sorted out (lines 86-104)*
In lines 100 - 101 you wrote: "third, Algeria was not captured, which implied that the study could not be applied to all the countries in the region". Do you review address this? Does your review capture all North African countries given the recent conflicts?

**The authors' response*:* yes**

**Review this sentence in lines 107 - 110 for grammatical error and break sentence in two: "The main goal of this study is to determine through a systematic review and meta-analysis of the prevalence of undernutrition (stunting, wasting, and underweight) among under-five children in North Africa and aimed to ascertain if those countries are on course to achieve the SDGs 1, 2, and 3 by the year 2030."**
**The authors' response*:* sorted out***.*  *Therefore, this systematic review and meta-analysis study was conducted to determine the prevalence of undernutrition (stunting, wasting, and underweight) among under-five children, specifically and thoroughly, in the North African region. As a result of this systematic review, we will also be able to determine whether those countries are on track to meet SDGs 1, 2, and 3 by 2030. (Lines 111-116).*

**Method
What is the difference between lines 135 - 136: "Furthermore, Google Scholar and the reference lists of retrieved articles were screened for further relevant publications." and lines 140 - 143: "Furthermore, to avoid missing any additional relevant publications, we searched the bibliographical references of all retrieved articles that met the inclusion criteria, complemented by citation tracking by the use of Google Scholar". In my view, both sentences are communicating the same message. So I recommend you remove one of them.**
**The authors' response*:*** *sorted out and the second sentence was deleted (line 140)*

**Why were grey literature not included? Given the situation in some countries in North Africa, some government or non-governmental organizations may have some useful documents on child undernutrition in their individual countries.
The authors' response*:*** *Firstly, our eligibility criteria were to include only* ***peer reviewed articles*** ***written in*** ***English****; accordingly, no grey literature were results from our 5 targeted databases to be included. Secondly, data in identified literature was taken from government websites and reports, so we do not want to duplicate work. For example, the Egypt Demographic and Health Survey (DHS) 2014 (report) by the Ministry of Health and Population Cairo, Egypt, this survey is used as the main source of national data by Almasi et al. 2019, Figueroa and Kurdi 2019, Ghattas et al. 2020, Shaker-Berbari et al. 2021, Sharaf and Rashad 2016. Data from the DHS 2005, 2003, 2000, 1995, and 1992 was used by Tzioumis et al. 2016, and those from the DHS 2000 and 2008 were used by Ozaltin et al. 2010. El Taguri 2008 and 2009 used MICS (1995, 2003), and Almasi et al. 2009 used PAPFAM 2007, the three available national representative surveys in government reports and organisations' websites for Libya. Morocco national surveys in* 2004, 1997, and 1992 *have been taken from the Ministry of Health national reports, as stated by Barouaca 2012. Ozaltin et al. 2010 and Eltaguri 2009 also reported data from the Moroccan national survey in 2003.*

*However, due to our systematic study inclusion criteria by including only studies written in the English language (which was already stated as one of our study limitations), we could not include many studies targeted Algeria because Algeria's national reports, surveys, and studies usual uses French language and some have Arabic language copy, too especially in Algerian government websites, so we could not get enough studies as they almost written by languages rather than English.*

- [*https://mics-surveys-prod.s3.amazonaws.com/MICS6/Middle%20East%20and%20North%20Africa/Algeria/2018-2019/Survey%20findings/Algeria%202018-19%20MICS_French.pdf*](https://mics-surveys-prod.s3.amazonaws.com/MICS6/Middle%20East%20and%20North%20Africa/Algeria/2018-2019/Survey%20findings/Algeria%202018-19%20MICS_French.pdf)
- [Facteurs associés à l’âge d’initiation de la diversification alimentaire chez des enfants âgés de 6 à 60 mois dans l’est algérien : corrélation à l’état nutritionnel - ScienceDirect](https://www.sciencedirect.com/science/article/abs/pii/S0985056220304441?via%3Dihub)

**Results
Include in Table 1 the prevalence of stunting, wasting and underweight as reported in the individual studies OR create a separate table that outlines the prevalence of stunting, wasting and underweight which informs the forest plots.
The authors' response*:*** This table is already in the supporting information S4 Table. Since we already have busy graphs with table 1 (the characteristics of identified studies) in the main manuscript, we decided to leave it out as they already mentioned in the graphs.

**Discussion
In lines 274 and 275, replace the words "utilised" and "investigate" respectively with more suitable words.
*The authors' response****: sorted out (lines 283-284)*

**Review this comment in lines 281 - 283: "Accordingly, the overall pooled prevalence of stunting among the under-5 children in North Africa was approximately one in every five. This is higher than that of the global average of 22%. It is however lower than that of West Africa (30.9%)". If you intend making comparison, then use the same metrics. What % is "one in every five?".
*The authors' response:*** *sorted out (lines 291-292)*
**In lines 370 - 371, how does excluding hospitalized children enhance the generalizability of results to the general population?**

***The authors' response*:** The study eligibility criteria excluded studies that investigated or targeted hospitalized children that enhanced generalizability since hospitalized children already had health problems that could affect their nutritional health or weight. Thus, our results were not influenced by ill children and were generalizable to all children in the targeted community.
