# Supplementary material for: A systematic review and meta-analysis of the prevalence of childhood undernutrition in North Africa
Source: PLoS One. 2023 Apr 6;18(4):e0283685. doi: 10.1371/journal.pone.0283685 (PMC10079122; doi:10.1371/journal.pone.0283685)
Supplement: Attachment — Submitted filename: Response to Reviewers.docx [file pone.0283685.s010.docx]

**Response to editors**

12 March 2023

**Manuscript ID:** PONE-D-22-26443

“A systematic review and meta-analysis of the prevalence of childhood undernutrition in North Africa” (**Authors:** **Elmighrabi, Fleming, Dhami, Elmabsout & Agho**)

**Dear Editor-in-chief,**

Thank you for allowing us to revise and re-submit our manuscript. We are incredibly grateful for your insightful feedback that has strengthened our manuscript.

We addressed all comments raised by the editor in the revised manuscript. Editing changes made to the revised manuscript have been highlighted in yellow. One of the paper's authors, who speaks English as a first language, has reviewed the paper.

Below, we provide a point-by-point response to the comments and, where possible, linked our responses to the appropriate sections in the revised manuscript. We hope that the revisions made have properly addressed the concerns and that our revised manuscript is accepted for publication in the journal.

*Our open-access publication fees have already been approved, but they are only valid for a limited time. It would be great if you could get back to us soon, so we don't lose this opportunity*.

**Response**

1- **because of: (line 55)”**

**Authors response:** sorted out (line 54)

2- **“Development, (line 62)”**

**Authors response:** sorted out (line 64)

3- **Another a recent review (line 86)”**

**Authors response:** sorted out (line 82)

4- **“(23,24); Fourthly,”**

**Authors response:** sorted our (line 93)

5- **” Table1 (line 168)”**

**Authors response:** sorted out (line 171)

6- **“Lybia 4.3 (line 276)**

**Authors response:** sorted out (line 254)

7- **“in in (line 312)”**

**Authors response:** sorted out (line 284)

8- **so on**

**Authors response: We conducted a thorough English editing process to improve the paper’s readability and clarity.**

9- **Break sentences to improve readability (Line 91-98)**

**Authors response:** sorted out (line 82-95)

10- **check the reference format. For example, Reference #57**

**Authors response:** sorted out (Reference 55); we thoroughly reviewed and checked all references.
